# Supplementary material for: Complexome profiling of the Chlamydomonas psb28 mutant reveals TEF5 as an early PSII assembly factor
Source: Plant Cell. 2025 Mar 17;37(6):koaf055. doi: 10.1093/plcell/koaf055 (PMC12164590; doi:10.1093/plcell/koaf055)
Supplement: koaf055_Supplementary_Data [file koaf055_supplementary_data.zip › psb28_interactive.zip!/plots/intensity/qualityPlots/peptides/ACK2_Cre17.g709850.t1.2.html]

### Cre17.g709850.t1.2

Trivial name: ACK2  
SeqCoverage [median]: 8.20  
Mass: 43.50 kDa  
  
 

| Sequence | MissedCleavage | Mass [Da] | wtIntensity | muIntensity | Score |
| --- | --- | --- | --- | --- | --- |
| AGVISTPGSR | 0 | 943.51 | 851.40 | 0.00 | 71.50 |
| AVIEGAGKGELR | 1 | 1198.67 | 839.32 | 0.00 | 67.46 |
| VLVLNAGSSSLK | 0 | 1186.69 | 0.00 | 1419.51 | 64.04 |
|  |  |  | 1690.7 | 1419.5 |  |
